# Graham's Patch Versus Modified Graham's Patch in the Management of Perforated Duodenal Ulcer

**DOI:** 10.7759/cureus.96486

**Published:** 2025-11-10

**Authors:** Shahper Reza, Md Arifur Rahman, Zain Girach, A B M Monirul Islam, Maj Musarrat Tasneem, M Samia Islam

**Affiliations:** 1 Department of Surgery, Holy Family Red Crescent Medical College Hospital, Dhaka, BGD; 2 Department of General Surgery, Kettering General Hospital NHS Foundation Trust, Kettering, GBR; 3 Department of Anesthesiology, Combined Military Hospital, Dhaka, BGD; 4 Department of Oral and Maxillofacial Surgery, Dhaka Dental College and Hospital, Dhaka, BGD

**Keywords:** complicated peptic ulcer disease, graham's patch, modified graham's patch, perforated duodenal ulcer, surgical outcome

## Abstract

Introduction: Perforation of a duodenal ulcer is a severe complication of peptic ulcer disease that requires prompt resuscitation and appropriate surgical intervention to minimize morbidity and mortality. This study aimed to compare the outcomes and complications of Graham's patch versus modified Graham's patch repair in patients with perforated duodenal ulcers admitted to a tertiary care hospital in Bangladesh.

Methods: This comparative observational study was conducted in the Department of Surgery at Dhaka Medical College Hospital (DMCH) in Dhaka, Bangladesh, over a six-month period in 2018. Patients diagnosed with duodenal ulcer perforation were selected based on pre-defined inclusion criteria. Informed written consent was obtained from all participants prior to enrollment. A total of 60 patients (30 in each group) were included using a systematic sampling method. Data on post-operative outcomes and complications, such as leakage and wound infection, were collected using a pre-designed data sheet. Data analysis was performed using IBM SPSS Statistics for Windows, V. 24.0 (IBM Corp., Armonk, NY, USA).

Results: The study included 60 participants with a mean age of 34.22±8.59 years (range: 16-53 years) and a male-to-female ratio of 2:1. In the Graham's patch group, 90% of patients had pus in the intraperitoneal cavity, compared to 67% in the modified Graham's patch group. Wound infection and post-operative fever were the most common complications observed in both groups, with no statistically significant differences (p=0.59; p=0.78). The average hospital stay was six days for the Graham's patch group and eight days for the modified Graham's patch group, also showing no significant difference between the groups (p=0.145).

Conclusion: This study compared the short-term outcomes of traditional versus modified Graham's patch repairs for perforated duodenal ulcers and found no significant differences in post-operative complications or hospital stay. Both techniques were safe and effective, allowing surgeons to choose based on experience and intra-operative factors, with the modified patch potentially offering easier handling for certain cases. However, due to limitations like small sample size and short follow-up, larger multi-center trials are needed to better assess long-term outcomes and guide surgical practice.

## Introduction

Perforation is one of the most severe complications of peptic ulcer disease (PUD), occurring in approximately 2-10% of affected individuals [1]. The condition carries a significant risk of death, with overall mortality estimated at around 10%, though studies have reported rates ranging from 1.3% to as high as 20% [1-6]. Among all complications linked to peptic ulcers, perforation is the most frequently encountered. It most commonly occurs at specific anatomical locations: about 60% of cases involve the anterior wall of the first portion of the duodenum, while 20% are found in the gastric antrum and another 20% along the lesser curvature of the stomach [7]. Perforation occurs when the ulcer erodes through the full thickness of the stomach or duodenum. The occurrence of perforated PUD has been strongly associated with bleeding ulcers and the use of non-steroidal anti-inflammatory drugs (NSAIDs) or aspirin, particularly among the elderly. Studies indicate that more than one in five patients over 60 years old who experience a perforated ulcer are actively using NSAIDs at the time of the event [8].

The standard surgical procedure for addressing a perforated ulcer is known as Graham's patch repair, first detailed by Roscoe Graham in 1937. In this technique, once the abdomen is opened via laparotomy and the perforation is located, three to four stitches are placed around the edge of the ulcer. Before these sutures are tied off, a nearby portion of the omentum (a fatty layer of tissue) is positioned over the perforation. The omentum is gently laid across the anterior surface of the duodenum and secured as the sutures are tied in sequence from top to bottom, effectively sealing the hole with the omental tissue. It is crucial that the sutures hold the omentum firmly without constricting it, as this would reduce blood flow and compromise the tissue's viability. The patch must remain healthy and well-perfused to ensure proper healing [9].

An adaptation of this method, referred to as the modified Graham's patch repair, follows a slightly different approach. In this version, the ulcer is first closed with the initial set of sutures. Afterward, the omentum is placed over the closed perforation and secured with a second set of knots. However, this method has raised concerns that the omentum might not adhere as closely to the site, potentially making the seal less effective than in the traditional method where the omentum is applied directly to the open ulcer [10].

Possible complications after surgery include pneumonia, infections at the surgical site, abdominal abscesses, and cardiovascular complications. Other post-operative issues may involve diarrhea in up to 30% of cases following vagotomy and dumping syndrome in about 10% of those who undergo vagotomy with drainage procedures [11].

A perforated duodenal ulcer is very common in Bangladesh. There is a lack of studies exploring the different methods of surgical repair of a perforated duodenal ulcer. The current study aimed to contribute to identifying the outcome and complications of Graham's patch and modified Graham's patch repair in perforated duodenal ulcer. 

## Materials and methods

This comparative observational study was conducted in the Department of Surgery at Dhaka Medical College Hospital, Dhaka, Bangladesh, over a six-month period in 2018. The study population included patients who presented with an acute abdomen to the surgery ward during this time. A systematic sampling method was employed, where every alternate patient was selected for inclusion in each of the two treatment groups. Specifically, the first patient and every alternate patient thereafter were assigned to undergo the Graham's patch procedure, while the second patient and every alternate patient thereafter underwent the modified Graham's patch procedure. 

The inclusion criteria included patients with suspected perforated chronic duodenal ulcer irrespective of age and sex. The exclusion criteria included patients with perforation older than five days, those with an American Society of Anesthesiologists (ASA) score ≥IV (indicating poor general condition), and patients with a Mannheim Peritonitis Index (MPI) >21 indicating extensive peritonitis, gastric perforation, and traumatic (stab, blunt, instrumental) perforation.

In this study, Graham's patch repair is defined as a surgical technique in which three to four sutures are placed around a perforation, and then a piece of free omentum is laid over these sutures to plug the perforation, after which the sutures are tied. The modified Graham's patch repair is defined as three to four sutures first placed and tied to close the perforation. Next, the omentum is positioned over the sutures that have already been tied, and an additional set of knots is used to secure the omentum in place. Post-operative leakage is identified by symptoms and signs such as tachycardia, fever, tachypnea, left shoulder pain, abdominal pain, chest pain, and the presence of bile in the drain and confirmed, where indicated, by contrast-enhanced CT imaging. An intra-abdominal abscess is defined as a collection of pus or infected fluid surrounded by inflamed tissue inside the abdominal cavity. Wound infection is indicated by the presence of pus or an abscess, fever with tenderness of the wound, or the separation of the edges of the incision exposing deeper tissues. Length of hospital stay is measured from the first post-operative day to the day of discharge from the hospital. A 30-day readmission refers to a subsequent hospital admission to the same hospital within 30 days following an initial admission.

A total of 60 patients were enrolled and divided into two groups, each consisting of 30 patients. One group underwent the traditional Graham's patch repair, while the other underwent the modified Graham's patch repair. A detailed clinical history was taken from each patient, including information on risk factors and comorbid conditions. All patients underwent hematological investigations including a complete hemogram, serum electrolytes, blood urea, serum creatinine, and blood sugar. Imaging studies such as abdominal ultrasound and erect abdominal X-ray were also performed. Pre-operative resuscitation involved the administration of crystalloids at a dose of 20-40 ml/kg of body weight to ensure good urinary output and stable hemodynamics. All patients received broad-spectrum antibiotics and metronidazole pre-operatively. Both surgical procedures were carried out under general anesthesia. Post-operative outcomes were recorded using a pre-designed proforma, and the collected data were analyzed using IBM SPSS Statistics for Windows, V. 24.0 (IBM Corp., Armonk, NY, USA). Exploratory data analysis was carried out to describe the study population where categorical variables were summarized using frequency tables, while continuous variables were summarized using measures of central tendency and dispersion such as mean, median, percentiles, and standard deviation. In order to determine associations, chi-squared tests were used. In each analysis, p-values of <0.05 were considered statistically significant.

Written informed consent was obtained from all patients prior to their participation in the study. Ethical considerations were carefully observed throughout the research process. Formal ethical approval was obtained from the Ethical Review Committee of Dhaka Medical College (approval number: MEU-DMC/ECC/2018/44). Confidentiality of personal and medical information was strictly maintained, and unauthorized individuals had no access to the data. 

## Results

Among 60 participants with perforated duodenal ulcers, there were two groups consisting of 30 patients each. One group received Graham's patch repair as their management, while the other group received the modified Graham's patch repair. 

Out of 60 patients, there were 40 (67%) male patients and 20 (33%) female patients. In the Graham's patch group, 60% (n=18) were male, and in the modified Graham's patch group, 73% (n=22) were male.

**Figure 1:**
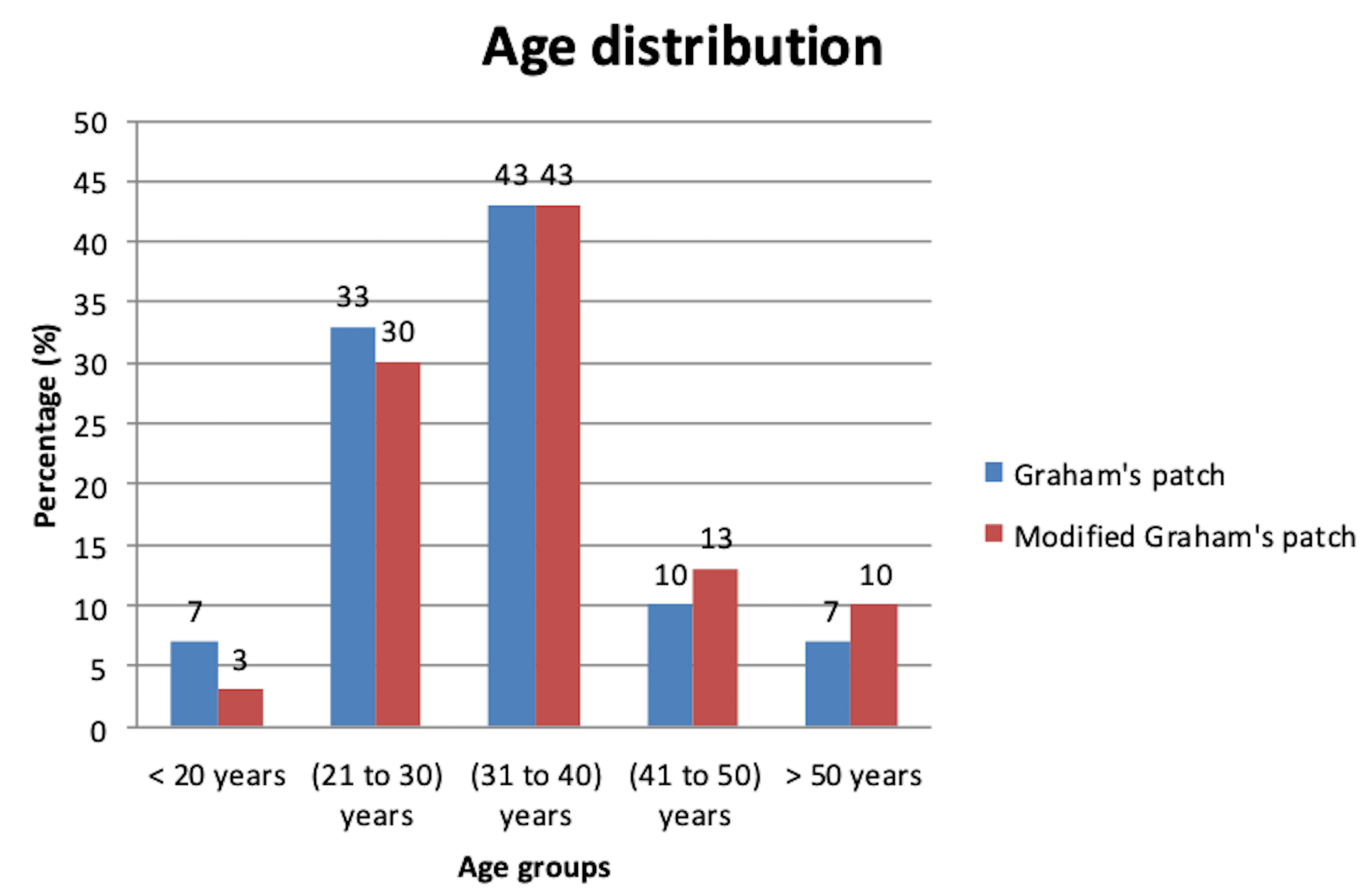
Distribution of perforated duodenal ulcer patients according to their age (n=60)

Out of 60 patients with perforated duodenal ulcer, the majority were from the age group of 31-40 years (Figure 1). The mean age of the patients was 34.22±8.59 years. The minimum age of the patients was 16, and the maximum age of the patients was 53.

Clinical presentations such as abdominal pain, nausea/vomiting, constipation, anemia, elevated temperature, rebound tenderness, and obliteration of liver dullness were collected for all patients with perforated duodenal ulcer pre-surgery. In both groups, abdominal pain, rebound tenderness, nausea/vomiting, and elevated temperature were found to be extremely prevalent (Table 1).

**Table 1:** Clinical presentation among patients in both groups *p<0.05 considered statistically significant

Symptoms	Graham's patch (n=30) %	Modified Graham's patch (n=30) %	P-value
Abdominal pain	29 (97)	29 (97)	1.00
Nausea/vomiting	25 (83)	26 (84.5)	0.89
Constipation	20 (67)	11 (37)	0.02*
Anemia	19 (63)	21 (70)	0.57
Elevated temperature	24 (80)	22 (73)	0.54
Rebound tenderness	26 (87)	24 (80)	0.49
Obliteration of liver dullness	18 (60)	15 (50)	0.42

Considering the variable size of duodenal perforation, the 0.6-1 cm group was most common among all patients (Table 2).

**Table 2:** The variable size of duodenal perforation

Size of perforation	Graham's patch (n=30) %	Modified Graham's patch (n=30) %	P-value
<0.5 cm	6 (20)	5 (16.7)	0.73
0.6-1 cm	20 (66.7)	20 (66.7)	1.00
>1 cm	4 (13)	5 (16.7)	0.71

The majority of patients in both groups had pus in the intraperitoneal cavity: 90% (n=27) in the Graham's patch group and 67% (n=20) in the modified Graham's patch group, respectively (Table 3).

**Table 3:** Distribution of patients according to presence of pus in the intraperitoneal cavity *p<0.05 considered statistically significant

Presence of pus	Graham's patch (n=30)	Graham's patch %	Modified Graham's patch (n=30)	Modified Graham's patch %	P-value
Present	27	90	20	67	0.03*
Absent	3	10	10	33	-

Wound infection and pyrexia were the most common post-operative complications among perforated duodenal ulcer patients in both groups. However, no significant (p<0.05) association was found for any complication (Table 4).

**Table 4:** The post-operative complications of both techniques P-value is determined by the chi-squared test

Name of complication	Graham's patch (n=30) %	Modified Graham's patch (n=30) %	P-value
Wound infection	9 (30)	12 (40)	0.59
Post-operative leakage	1 (3.3)	1 (3.3)	1.00
Intra-abdominal abscess	2 (7)	0 (0)	0.24
Pyrexia	10 (33)	8 (27)	0.78
Re-operation	2 (7)	1 (3.3)	0.67
Hospital stay (mean±SD, days)	6.12±2.34	7.78±1.39	0.145

## Discussion

Peptic ulcer perforation is a life-threatening complication of PUD. Currently, the most accepted method of surgical closure of the perforation is called Graham's patch repair. This method involves placing three to four sutures around a perforated ulcer, positioning a piece of the omentum over it, and tying the sutures to secure the omentum in place. The sutures must hold the omentum firmly without restricting its blood supply, ensuring the patch remains viable. This technique was later modified and called the modified Graham's patch repair in which the initial three to four sutures are tied to close the ulcer first and then an omental patch is placed over the closure and secured with a second set of knots. However, this method may not seal as effectively, as the omentum is not laid directly on the open ulcer bed [7-10,12].

In this study, a total of 60 patients with perforated duodenal ulcers were included in the final analysis. The patients were managed using two different surgical techniques: Graham's patch repair and the modified Graham's patch repair. They were evenly divided into two groups, with 30 patients in each group. The majority of patients in both groups (43%; n=13) were aged 31-40 years. The patients' ages ranged from 16 to 53 years, with a mean age of 34.22±8.59 years.

In the Graham's patch group, 60% (n=18) were male, while in the modified Graham's patch group, 73% (n=22) were male. This male predominance is consistent with findings from a cross-sectional study by Ekka and Malua at the Rajendra Institute of Medical Sciences, Ranchi, India, where 78.02% of patients were male and 21.98% were female, supporting the results of this study in showing the epidemiological characteristics of the perforated duodenal ulcer population [13].

Out of 60 patients with peptic ulcer perforation treated by Graham's patch repair and modified Graham’s patch repair, the percentage of clinical presentations are as follows: the majority (97%; n=27) in both groups presented with abdominal pain, followed by nausea/vomiting (83% (n=25) and 84.5% (n=26)) and rebound tenderness (87% (n=26) and 80% (n=24)). Therefore, similar baseline clinical presentations between the groups aid in reducing bias.

Among 60 patients with duodenal perforation, the most common perforation size was 0.6-1 cm among both groups (67%; n=20). Again, the similar severity of perforation between the two treatment groups aids in making a reliable comparison between them. Two further studies by Jat and Satapathy et al. also reported that 0.6-1 cm size duodenal perforation was found in the majority of cases [14,15]. These findings support our study showing our patient groups' severity of duodenal perforation is representative of other populations. 

In this study, the majority of patients (90% (n=27) and 67% (n=20)) had pus in the intraperitoneal cavity in the Graham's patch group and the modified Graham's patch group, respectively. Kidwai and Ansari in a similar study reported that the Graham's patch repair group had pus in the intraperitoneal cavity in 93.3% cases, while the modified Graham's patch repair group had pus collection in the intraperitoneal cavity in 73.3% cases [16]. These findings, in correlation with ours, show our study's groups to be representative of other duodenal perforation population groups; however, the severity of perforation in the Graham's patch group could be seen to be greater.

Out of 60 patients with perforated duodenal ulcer in this study, wound infection and pyrexia were the most common post-operative complications in both groups. Furthermore, between both groups, the prevalence of complications (wound infection, pyrexia, post-operative leakage, intra-abdominal abscess) was very similar, with no significant (p<0.05) association being found. 

In comparison with other studies, Ekka and Malua and Chalya et al. also reported supportive findings with the main post-operative complication being wound infection in both treatment groups [13,17]. However, both studies also reported chest/pulmonary infection as a prevalent complication, something that was not recorded in our study.

Among 60 perforated duodenal ulcer patients, everyone had to stay from five days to 14 days in the hospital. The mean value was six days for the Graham's patch group and eight days for the modified Graham's patch group; however, no statistical significance was found. Satapathy et al. also reported a greater mean hospital stay of 10.68 days vs 11.35 days in the Graham's patch group compared to the modified Graham's patch group [15]. The hospital stay varies with the size of the perforation, duration of illness, and condition of the patient on arrival.

Graham's patch repair and the modified version show comparable outcomes in terms of morbidity and mortality. Therefore, there is no statistically significant difference between the two techniques regarding patient recovery and survival rates. Either procedure can be performed based on the surgeon's preference. While the modified Graham's patch repair may be more technically convenient and surgeon-friendly for perforations in the first part of the duodenum, it requires careful execution. Proper caution must be taken during closure to ensure that the sutures are not too tight, which could cause tissue damage, nor too loose, which could lead to recurrence. The goal is to securely position the omentum, allowing it to adhere to the inflamed serosa and effectively seal the perforation [15-17].

This study was conducted at a single center, which may limit the generalizability of the findings. Additionally, long-term follow-up was beyond the scope of the study, and the sample size was relatively small, which may affect the statistical power and robustness of the conclusions.

## Conclusions

The present study evaluated the short-term outcomes of two commonly used surgical techniques for repairing perforated duodenal ulcers: the traditional Graham's patch and the modified Graham's patch repair. While post-operative outcomes such as leakage, intra-abdominal abscess, wound infection, and hospital stay did not differ significantly between the traditional Graham's patch and the modified Graham's patch groups, a significant baseline difference was noted in the presence of intraperitoneal pus (90% vs 67%), indicating that the groups were not fully comparable.

Therefore, the findings should be interpreted with caution. Both procedures appear safe when performed with proper surgical technique; however, due to baseline bias and limited sample size, the study cannot conclusively determine equal efficacy. Larger, multi-center randomized trials with standardized baseline assessment are needed to establish definitive recommendations for the surgical management of duodenal ulcer perforation.
